# Subacute Bilateral Subdural Hematoma: Delayed Presentation With Headache One Month Post Mild Trauma

**DOI:** 10.7759/cureus.19183

**Published:** 2021-11-01

**Authors:** Abdulrahman Alsahan, Mishael Alobaid, Talal Aldukhayyil, Dunya Alfaraj

**Affiliations:** 1 Medicine, Imam Abdulrahman Bin Faisal University, Khobar, SAU; 2 Emergency, Imam Abdulrahman Bin Faisal University, King Fahad University Hospital, Dammam, SAU

**Keywords:** computed tomography (ct ), headache, mild trauma, subdural hematoma (sdh), subacute

## Abstract

We herein report a case involving the development of a bilateral subacute subdural hematoma (SDH) after minor trauma, with only two wounds over the nose and no abnormal clinical and radiological findings at first presentation. A 25-year-old male patient presented to the emergency department (ED) after a minor trauma. X-ray was done on the facial bone to rule out nasal fracture which showed no abnormalities and then he was subsequently discharged. Three weeks later, the patient complained of a headache that persisted for a week, which brought him to the hospital. The initial impression was migraine after the primary healthcare visit, for which MRI was arranged, but as the headache persisted, he went to the ED twice again, and a CT scan was done during his second visit to the ED, which showed bilateral subacute SDH (SASDH).

## Introduction

Subdural hematoma (SDH) is the result of bleeding over the surface of the brain, beneath the dura. This condition can be acute, subacute, or chronic. Acute SDH is usually caused by severe and high-impact injuries [1]. Subacute subdural hematoma (SASDH) is known as the gradual pooling of blood in the subdural space that occurs in the period of 4-21 days from the head injury. Usually, it is caused by trauma. This collection causes compression on the brain which leads to the production of localized neurological manifestations, increased intracranial pressure, or altered level of consciousness.

Chronic SDH occurs after 21 days of head insult. It can be caused by trauma, intracranial hypotension, and defective coagulation [2]. A lot of risk factors can also contribute to this condition such as old age, alcoholism, certain medication such as antiplatelet, anti-coagulants, and lastly, previous brain injury [3].

This case report describes a patient with delayed presentation of bilateral subacute SDH (SASDH) who did not show any radiological or clinical signs at the time of initial presentation. The patient is medically free and not on any medication.

## Case presentation

**Figure 1:**
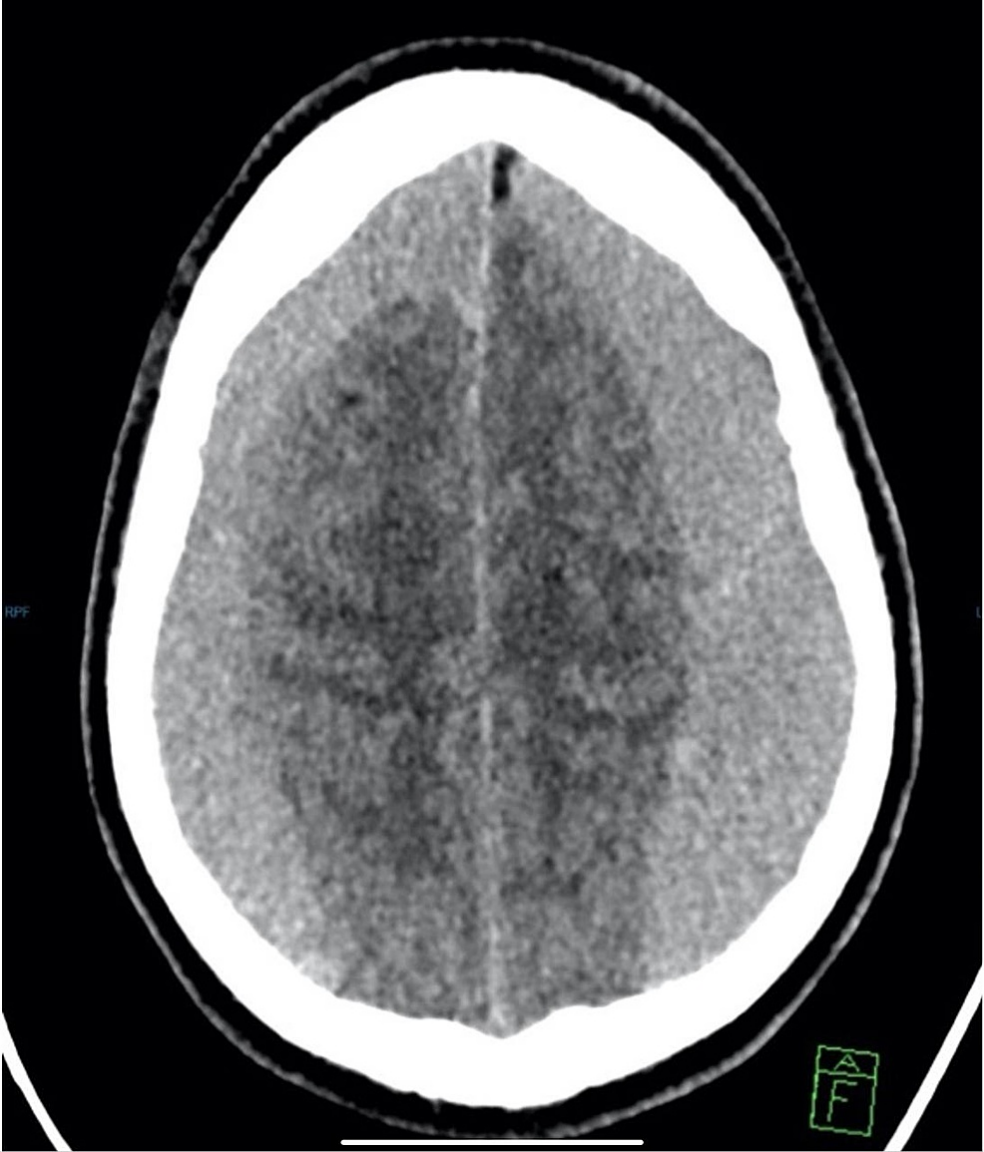
CT brain axial view: significant bilateral subacute subdural hematoma with significant mass effect on the brain cortex with effacement of the sulci and brain edema.

**Figure 2:**
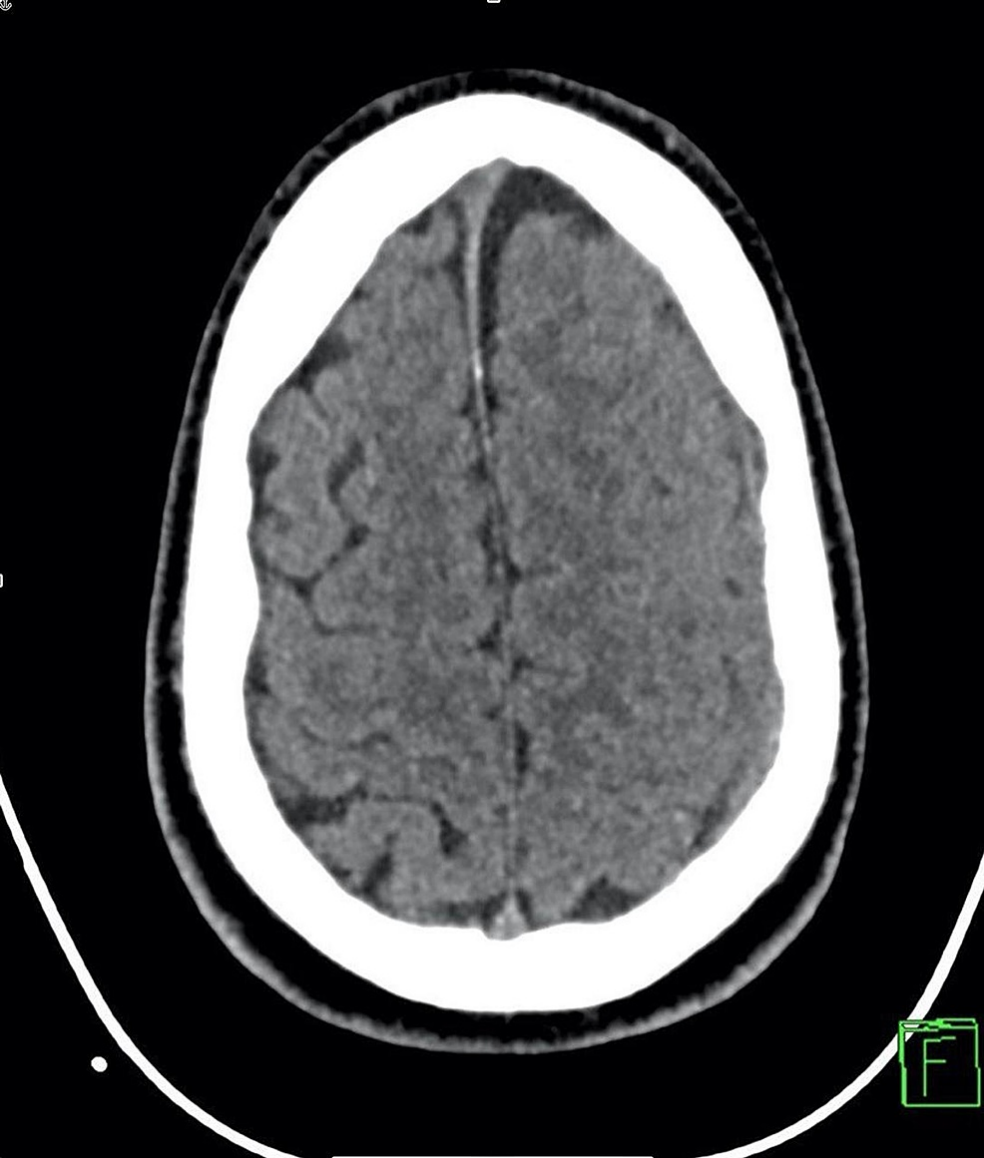
CT brain axial view: minimal left chronic residual subdural hematoma with no significant mass effect or midline shift.

A 25-year-old male who is medically free presented to the ED after a motor vehicle collision (MVC) with a speed of 50 m/h, frontal impaction and there was no roll over, or ejection from the car with minimal epistaxis and a small superficial wound over the nose. A nasal X-ray was done to rule out facial bone fractures, which shows no abnormality. Analgesia was given and he was referred to the otolaryngology department for follow-up. However, at that time CT scan was not done. He presented three weeks later to the primary healthcare in which the first impression was migraine and was given analgesia. However, as the headache persisted, he went to the ED and he was given analgesia and discharged. The patient returned to the ED two days later, because the headache was not gone. He described it as a throbbing headache, on and off, which increased with laying down, stress, and at night and relieved by analgesia. He scored the pain severity as nine out of 10 and described it as a pain he cannot tolerate. There was no loss of consciousness, seizures, blurred or double vision, weight loss, fever, weakness, or any neurological deficits. He was vitally stable, and the Glasgow Coma Scale (GCS) score was 15, oriented to time, place and person, intact memory, pupils reactive to light equally, all limbs power were five out of five, no pronator drift, intact sensation, and normal gait. At this time, the CT scan was done and showed bilateral frontoparietal SASDH with midline shift with mass effect upon the adjacent brain parenchyma (Figure 1). A cerebral angiogram with embolization was done for him. The patient improved after the intervention and was discharged. He followed up with neurosurgery and another CT which showed that the hematoma regressed in size. After this regression, conversion to chronic SDH was observed in CT (Figure 2).

## Discussion

Subacute SDH is usually caused by head trauma. The pathophysiology of SASDH is poorly understood. Thus, it is not easy to differentiate between SASDH and chronic SDH [4].

Subacute SDH is a result of subdural effusion that occurs in one to three weeks. A previous article mentioned that SASDH occupies the intradural space. They also mentioned that “Blood in this space provokes an inflammatory reaction, which results in an enveloping membrane surrounding the blood.” This is followed by ingrowth of neocapillaries, enzymatic fibrinolysis, and liquefaction of blood clots. The clinical presentation of patients with SASDH varies from asymptomatic to hemiparesis, loss of consciousness, headache, and seizure [5]. Rapid neurological deterioration usually distinguishes it from the chronic onset [6]. The delayed presentation of SASDH is rare. There are few studies that reported delayed presentation of SDH after trauma. One with subacute and two with chronic (CSDH). 

The first case was in 2015 of an 18-year-old male who presented to the ED four days after a minor trauma. The patient had a continuous headache since the trauma. The CT of the brain did not show any abnormalities and was discharged. The headache persisted and the patient returned to the ED two days after the last visit. The patient reported having a severe headache, nausea, and vomiting. A CT was performed, and it showed a left SDH. The patient was treated conservatively and was discharged. Eight days after the last visit, the patient presented with vomiting and a headache. Another CT was performed and revealed a left mixed dense SDH with a midline shift. The patient was diagnosed with SASDH and was treated with hematoma irrigation with trephination immediately. The patient completely recovered [7].

Another case reported in 2020 was that of an 84-year-old male, who presented eight hours post-injury to the ED with vomiting and dizziness. He was not on anticoagulants or antiplatelet. CT did not show any intracranial pathology or skull fractures. The patient was discharged after a 12-hour observation. Two months later, the patient presented to the same ED with dizziness and unsteady gait. CT was performed and showed a large CSDH that was located in the right side, compressing the third and right lateral ventricles. An emergency decompression with dual burr‐holes was performed. CT was performed later on and showed a reduction of mass effect and no hydrocephalus 48 hours post-surgery [3].

Another case back in 2005 was of a 56-year-old male, who complained 40 days after a minor trauma of neck pain in the right side and shoulder stiffness. CT revealed subdural effusion on the right side of the brain. He was followed up one, two, four, and six months after the first CT. The patient received no medical treatment during the follow-up. Nine months post-trauma, the patient represented with progressive head heaviness and left motor weakness. A CT was performed and showed a developed chronic subdural that was located on the right side and causes a slight shift to the left. Burr-hole surgery was performed, the hematoma was evacuated and irrigated with warm physiological saline solution, and a closed-system subdural drainage catheter was inserted. After the procedure, the patient showed no neurological signs and symptoms, and the operation appeared to be successful [8].

In comparison to the above cases, we found that all of the cases experienced a headache, but they were treated differently. Also, a CT scan was done in the first presentation after trauma in all of the cases. The first case was left-sided while the other two cases were right-sided. 

In our case, in the first presentation to the ED, a CT scan was not done. It was done at the third presentation. Although the SDH was bilateral and huge, the patient only presented with a headache. The above-mentioned cases present eight days, two months, and nine months after trauma respectively, while our case presented one month after the accident. Treatments used on this 25-year-old male patient were cerebral angiogram and embolization.

## Conclusions

The delayed presentation of bilateral massive SASDH without underlying pathology or major trauma is known to be rare. However, in the presented case with minor trauma even if the initial CT was negative, clear instructions about when to come back should be given to the patient. Also, the etiology of the presented case is unknown, so, it is recommended to conduct a genetic study for further understanding of the pathophysiology. In conclusion, physicians should take headache as a serious complaint post-trauma even if the trauma was mild and if the complaint was long after trauma.
